# Impact of dietary magnesium intake on depression risk in American adults: a cross-sectional study of the National Health and Nutrition Examination Survey 2005–2020

**DOI:** 10.3389/fnut.2025.1484344

**Published:** 2025-02-06

**Authors:** Yanping Huang, Su Ruan, Yang Yang, Hui Liang, Su Chen, Qing Chang

**Affiliations:** Department of Neurology, Affiliated People's Hospital, Fujian University of Traditional Chinese Medicine, Fuzhou, China

**Keywords:** depression, magnesium, mental health, cross-sectional study, nonlinear

## Abstract

**Introduction:**

Depression is a major global mental health challenge. Previous research suggests a link between magnesium consumption and depression, but the dose–response relationship remains unclear. This study investigates the relationship between dietary magnesium intake and depression risk among American adults.

**Methods:**

Data from the 2005–2020 National Health and Nutrition Examination Survey (NHANES) were examined. Depression was measured with the Patient Health Questionnaire-9 (PHQ-9), and dietary magnesium consumption was calculated from two 24-h meal recalls. We used restricted cubic spline models, logistic regression, and sensitivity analyses to assess the connection.

**Results:**

Among 35,252 participants (mean age: 49.5 ± 17.6 years; 49.9% women), we observed a nonlinearity in the relationship between dietary magnesium intake and depression. Below the inflection point (366.7 mg/day), the odds ratio (OR) was 0.998 (95% CI: 0.997–0.999, *p* < 0.001). Above this point, the OR was 1.001 (95% CI: 1.000–1.002, *p* = 0.007). In participants aged ≥60 years, the association was inverse L-shaped, with magnesium intake ≥270.7 mg/day increasing depression incidence by 0.1% per 1 mg/d increase.

**Conclusion:**

A nonlinear dose–response relationship exists between dietary magnesium intake and depression risk among US adults. Age significantly moderates this association, suggesting dietary recommendations should be tailored to different age groups.

## 1 Introduction

Depression is the leading cause of mental health-related impairment worldwide (1), affecting almost 300 million people. Depression reduces human capital, prevents people from realizing their full potential, and is linked to early death from illnesses and suicide. According to a recent analysis, there is a connection between depression and certain nutrients (2),which may be linked to either a decreased incidence of depression (3) or a higher chance of getting depression (4–6). In order to help prevent or treat depression, it is imperative to investigate other dietary components that may be linked to this illness.

Magnesium is the fourth most abundant element on Earth and a necessary cofactor for over 600 enzymes involved in several vital catalytic events, which play an important role in biological processes (7). Magnesium is generally obtained via the consumption of green leafy vegetables, whole grains, nuts, and fish. It is absorbed in the gastrointestinal and renal systems and helps with calcium (Ca^2^) absorption. Both ions are regulated by parathyroid hormone; however, free ion concentrations do not always match total concentrations (8). Magnesium is required for proper neurotransmission and is involved in the synthesis of membrane phospholipids, which play an important role in brain function and emotional regulation (9). Recent research (10, 11)have shown that nutritional habits have a considerable impact on mental health, particularly in stressful or challenging circumstances, implying that dietary patterns can influence psychological well-being. Magnesium’s antidepressant effects may be mediated through a variety of methods. The most prominent appears to be the inhibition of glutamatergic N-methyl-D-aspartate (NMDA) receptors (12); some mediation also occurs through serotonin system control (13). Interestingly, research in rats have demonstrated that magnesium-deficient diets are related with abnormalities in the gut microbiota, which eventually leads to disruptions in the gut-brain axis and the development of depression-like behaviors (14). This rising body of research emphasizes magnesium’s multidimensional involvement in mental health, as well as its potential as a modifiable dietary element for depression reduction.

Despite overwhelming evidence of magnesium’s role in mental health, the majority of published research focuses on qualitative linkages, with minimal investigation into the dose–response relationship between dietary magnesium intake and depression risk. This study analyzes data from the National Health and Nutrition Examination Survey (NHANES) to assess the relationship between dietary magnesium consumption and depression in the whole US population, including dose–response dynamics. These studies aim to provide new insights into the role of dietary magnesium in depression risk across various population subgroups.

## 2 Materials and methods

### 2.1 Data sources and study population

This cross-sectional analysis used data from the CDC’s 2005 ~ 2020 National Health and Nutrition Examination Survey (NHANES) (15). NHANES is a nationally representative survey that uses a stratified, multistage probability sampling design to assess the health and nutritional status of noninstitutionalized Americans (16). Data collection includes household interviews, physical examinations, and laboratory tests at mobile examination centers (MECs), ensuring comprehensive health assessments. The survey collects data on a wide range of variables, including demographic information, health conditions, lifestyle behaviors, and dietary intake, which are crucial for understanding health trends across the population. The ethical clearance for this study follows the protocols established by the NHANES program, which is conducted by the National Center for Health Statistics (NCHS). The NHANES procedures are reviewed and approved by the Ethics Review Board of the NCHS. All participants in NHANES provide written informed consent before participation, ensuring compliance with ethical guidelines. As our study is a secondary analysis of publicly available NHANES data, further Institutional Review Board (IRB) approval is not required (17). NHANES data is freely available on the NHANES website.^1^ Our study included interviewees aged 20 and over. We excluded pregnant women and individuals with missing data on the PHQ-9 questionnaire, dietary magnesium intake, and covariates.

### 2.2 Outcome ascertainment

Depression symptoms were assessed using the PHQ-9, a validated, dependable, and useful measure that combines the DSM-IV diagnostic criteria for depression and is appropriate for use in both clinical and research contexts (18). Participants rated each item based on their experiences in the 2 weeks preceding the questionnaire, with response categories scored as follows: 0 (not at all), 1 (a few days), 2 (more than half of the days), and 3 (almost every day). The total score varies between 0 and 27. In line with prior research, we classified the participants’ PHQ-9 scores as <10 (with no depression) or ≥10 (with depression) for this study (19).

### 2.3 Dietary assessment

Dietary data, including total energy, carbohydrate, and magnesium levels, were gathered during two 24-h dietary recalls. The first dietary recall interview was held at the Mobile Examination Center (MEC), and the second was completed over the phone after 3–10 days. Participants were asked to recall all foods and beverages ingested within the first 24 h before the interview (from midnight to midnight). Throughout both interviews, participants were given a set of measuring instructions and a food model booklet to help them report food quantities (20). The average values for total calories, carbohydrate, and dietary magnesium intake were computed using 24-h recall data. The 24-h dietary supplementation session included an interview on the use of nutritional supplements and over-the-counter antacids. The average daily consumption of dietary supplements was calculated by adding all supplemental nutrients and dividing by 30. Dietary magnesium intake was calculated using the average value, and total magnesium intake was defined as the sum of dietary magnesium intake and average daily supplement intake. Subjects were assigned to quintiles based on their dietary magnesium intake.

### 2.4 Covariates assessment

Based on the research (21–24), age, sex, marital status, race/ethnicity, education level, family income, smoking status, hypertension, diabetes, stroke, coronary heart disease, body mass index (BMI), caloric intake, and carbohydrate intake were all considered. Race and ethnicity were classified as White, Black, or Other. Marital status was defined as married, living with a partner, or living alone. There were two schooling levels: ≤12 years and >12 years. A US government research (25) categorized family income into three categories based on the poverty income ratio (PIR): low (PIR ≤ 1.3), intermediate (PIR > 1.3 to 3.5), and high (PIR > 3.5). According to prior research, the smoking status was classified as never smokers (fewer than 100 cigarettes smoked), current smokers, and former smokers (those who quit after smoking more than 100 cigarettes). Past ailments (hypertension, diabetes, stroke, and coronary heart disease) were determined using survey replies as to whether a doctor had ever told the participant about the condition. BMI was estimated using established methods based on weight and height.

### 2.5 Statistical analyses

The study was meticulously constructed in accordance with the STROBE standards (26). Categorical variables were presented as percentages (%), whereas continuous variables were presented as means (SD) or medians (IQR). To examine differences across groups, single-factor analysis of variance (for normally distributed data), Kruska-Wallis test (for skewed distributed data), and chi-square test (for categorical variables) were applied. Logistic regression models were used to calculate the odds ratios (OR) and 95% confidence intervals (95% CI) for the association between dietary magnesium consumption and depression. Model 1 accounts for sociodemographic factors such as age, gender, race/ethnicity, marital status, education level, and household income. Model 2 adjusted for covariates with *p*-values <0.05 in univariate analysis and sociodemographic parameters. Model 3 made a full adjustment for sociodemographic variables, smoking status, hypertension, diabetes, stroke, coronary heart disease, body mass index (BMI), calorie consumption, and carbohydrate intake.

Based on the variables adjusted in Model 3, restricted cubic spline (RCS) regression was performed with knots placed at the 5th, 35th, 65th, and 95th percentiles of dietary magnesium intake and divided into four segments to assess linearity and investigate the dose–response curve between dietary magnesium intake and depression.

We used a smooth two-piece logistic regression model to examine the threshold relationship between dietary magnesium consumption and depression after controlling for the factors in Model 3. The inflection point was determined using likelihood ratio tests and bootstrapping resampling.

We analyzed the relationship between dietary magnesium and depression according to gender, age (20–60 years vs. ≥60 years), education level (≤12 vs. >12 years), marital status (married or cohabiting vs. living alone), family income (low vs. medium or high), race, BMI (<25 vs. ≥25 kg/m^2^), smoking status, hypertension, diabetes, stroke, and coronary heart disease. Heterogeneity among categories was assessed using multivariable logistic regression, and the relationships between subgroups and dietary magnesium intake were investigated using likelihood ratio tests. We conducted sensitivity analyses by removing subjects with excessive energy consumption (<500 or >5,000 kcal/day) to evaluate the robustness of our findings.

Sample size was determined solely on the provided data; therefore, no *a priori* statistical power estimation was performed. All analyses were conducted using R version 4.2.2^2^ and Free Statistics software version 1.9.2. Descriptive statistics were performed for all participants. A two-tailed *p*-value of <0.05 was declared significant.

## 3 Results

### 3.1 Study population

**Figure 1:**
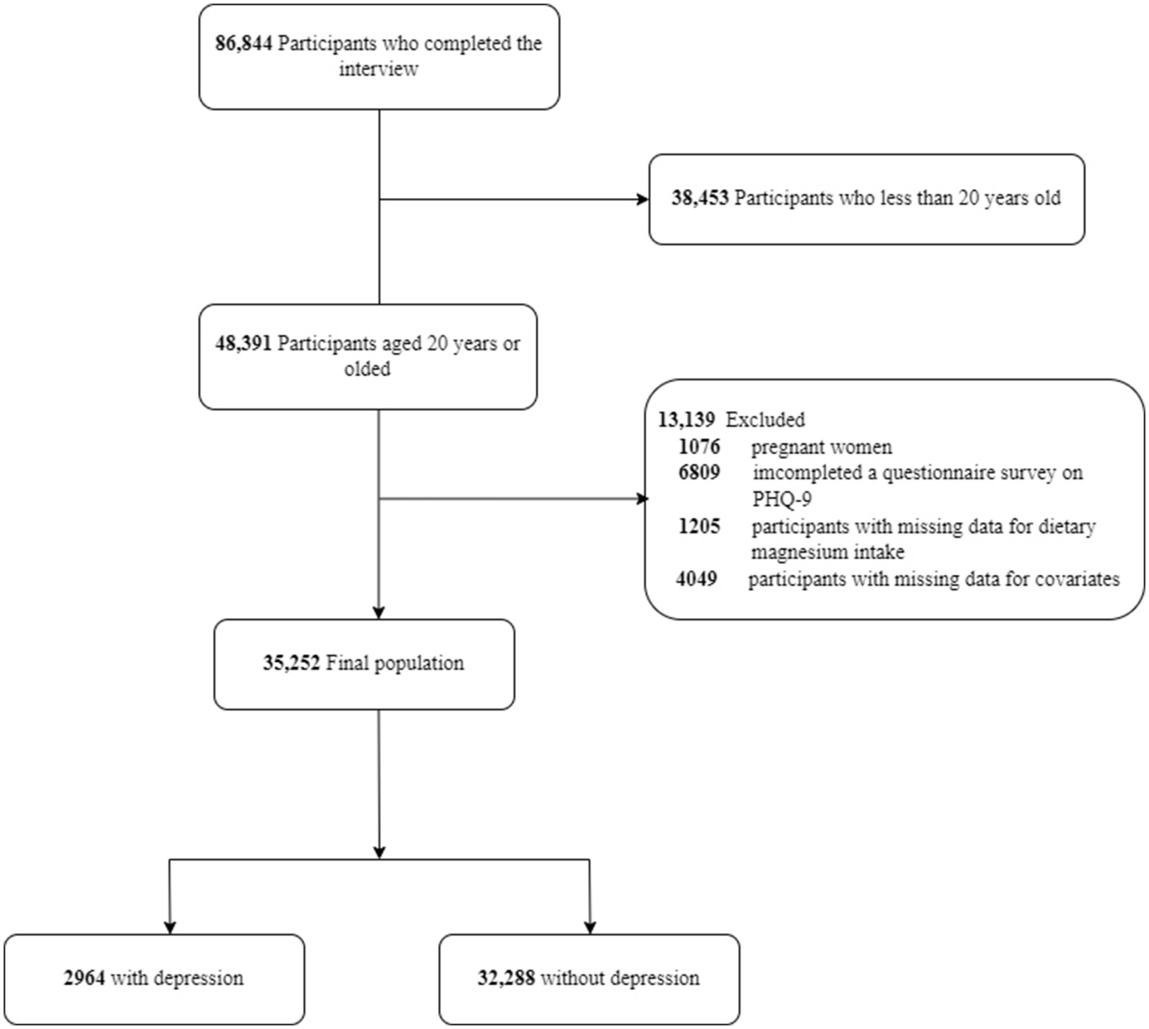
The flow chart of inclusion and exclusion of the participants in the study.

The interviews were completed by 86,844 individuals, 38,453 of whom were under the age of 20. Individuals who met the following criteria were excluded: pregnant women (*n* = 1,076); missing PHQ-9 questionnaire data (*n* = 6,809); missing dietary magnesium intake data (*n* = 1,205); and missing covariate data (*n* = 4,049). This cross-sectional study included 35,252 NHANES participants from 2005 to 2020. Figure 1 illustrates the detailed inclusion and exclusion processes.

### 3.2 Baseline characteristics

Table 1 presents the baseline characteristics of all participants by quartile of dietary magnesium consumption. A total of 2,964 people (8.4%) experienced depression. The average age of the participants was 49.5 (17.6) years, with 17,589 (49.9%) women and 17,663 (50.1%) men. Those who consumed more magnesium were more likely to be male, married or living with a partner, non-Hispanic White, never smokers, more educated; had a higher family income, a lower prevalence of hypertension, diabetes, stroke, and coronary heart disease and higher energy and carbohydrate consumption.

**Table 1:** Baseline characteristic of participants according to magnesium quartiles groups.

Characteristic	Magnesium (mg/d)						
	Total (*n* = 35,252)	Q1^d^ (*n* = 7,019)	Q2^e^ (*n* = 7,011)	Q3^f^ (*n* = 7,083)	Q4^g^ (*n* = 7,074)	Q5^h^ (*n* = 7,065)	*p*-value
Magnesium (mg/d)		≤ 192.4	(192.5 ~ 252.0)	(252.1 ~ 314.4)	(314.5 ~ 406.9)	≥ 407.0	
Age, Mean (SD^a^)	49.5 (17.6)	49.1 (18.6)	49.7 (18.1)	49.5 (17.7)	49.9 (17.1)	49.3 (16.5)	0.057
Sex, *n* (%)							<0.001^*^
Male	17,663 (50.1)	2,375 (33.8)	2,905 (41.4)	3,473 (49)	4,123 (58.3)	4,787 (67.8)	
Female	17,589 (49.9)	4,644 (66.2)	4,106 (58.6)	3,610 (51)	2,951 (41.7)	2,278 (32.2)	
Race/Ethnicity, *n* (%)							<0.001^*^
Non-Hispanic White	15,899 (45.1)	2,663 (37.9)	3,011 (42.9)	3,166 (44.7)	3,445 (48.7)	3,614 (51.2)	
Non-Hispanic Black	7,788 (22.1)	2,334 (33.3)	1805 (25.7)	1,491 (21.1)	1,170 (16.5)	988 (14)	
Others	11,565 (32.8)	2022 (28.8)	2,195 (31.3)	2,426 (34.3)	2,459 (34.8)	2,463 (34.9)	
Education level (year), *n* (%)							<0.001^*^
≤12	16,186 (45.9)	4,160 (59.3)	3,463 (49.4)	3,206 (45.3)	2,854 (40.3)	2,503 (35.4)	
>12	19,066 (54.1)	2,859 (40.7)	3,548 (50.6)	3,877 (54.7)	4,220 (59.7)	4,562 (64.6)	
Marital status, *n* (%)							<0.001^*^
Married or living with a partner	21,132 (59.9)	3,589 (51.1)	4,012 (57.2)	4,393 (62)	4,561 (64.5)	4,577 (64.8)	
Living alone	14,120 (40.1)	3,430 (48.9)	2,999 (42.8)	2,690 (38)	2,513 (35.5)	2,488(35.2)	
Family income, *n* (%)							< 0.001^*^
Low	10,509(29.8)	2,883(41.1)	2,194 (31.3)	2012 (28.4)	1749 (24.7)	1,671(23.7)	
Medium	13,391(38.0)	2,747 (39.1)	2,884 (41.1)	2,715 (38.3)	2,615 (37)	2,430 (34.4)	
High	11,352 (32.2)	1,389 (19.8)	1933 (27.6)	2,356 (33.3)	2,710 (38.3)	2,964 (42)	
BMI^b^ (kg/m^2^), Mean (SD)	29.3 (7.1)	30.0 (7.7)	29.9 (7.6)	29.3 (6.9)	28.9 (6.6)	28.5 (6.4)	<0.001^*^
Smoking status, *n* (%)							<0.001^*^
Never	19,123 (54.2)	3,683 (52.5)	3,905 (55.7)	3,954 (55.8)	3,832 (54.2)	3,749 (53.1)	
Current	8,778 (24.9)	1,431 (20.4)	1,611 (23)	1742 (24.6)	1951 (27.6)	2043 (28.9)	
Former	7,351 (20.9)	1905 (27.1)	1,495 (21.3)	1,387 (19.6)	1,291 (18.2)	1,273 (18)	
Hypertension, *n* (%)							<0.001^*^
No	24,882 (70.6)	4,768 (67.9)	4,809 (68.6)	5,041 (71.2)	5,109 (72.2)	5,155 (73)	
Yes	10,370 (29.4)	2,251 (32.1)	2,202 (31.4)	2042 (28.8)	1965 (27.8)	1910 (27)	
Diabetes, *n* (%)							<0.001^*^
No	30,002 (85.1)	5,852 (83.4)	5,862 (83.6)	6,041 (85.3)	6,070 (85.8)	6,177 (87.4)	
Yes	5,250 (14.9)	1,167 (16.6)	1,149 (16.4)	1,042 (14.7)	1,004 (14.2)	888 (12.6)	
Stroke, *n* (%)							<0.001^*^
No	33,928 (96.2)	6,617 (94.3)	6,724 (95.9)	6,826 (96.4)	6,874 (97.2)	6,887 (97.5)	
Yes	1,324 (3.8)	402 (5.7)	287 (4.1)	257 (3.6)	200 (2.8)	178 (2.5)	<0.001^*^
CHD^c^, *n* (%)							0.195
No	33,818 (95.9)	6,724 (95.8)	6,696 (95.5)	6,798 (96)	6,801 (96.1)	6,799 (96.2)	
Yes	1,434 (4.1)	295 (4.2)	315 (4.5)	285 (4)	273 (3.9)	266 (3.8)	
Depression, *n* (%)							<0.001^*^
No	32,288 (91.6)	6,144 (87.5)	6,381 (91)	6,521 (92.1)	6,636 (93.8)	6,606 (93.5)	
Yes	2,964 (8.4)	875 (12.5)	630 (9)	562 (7.9)	438 (6.2)	459 (6.5)	
Calorie consumption (kcal/d), Mean (SD)	2061.2 (862.7)	1332.1 (474.8)	1748.1 (511.2)	2033.2 (588.8)	2324.5 (675.1)	2860.6 (1045.1)	<0.001^*^
Carbohydrate consumption (g/d), Mean (SD)	247.9 (109.3)	167.1 (72.4)	211.9 (74.4)	245.5 (83.1)	278.2 (90.5)	335.9 (131.8)	<0.001^*^

SD^a^, standard deviation; BMI^b^, body mass index; CHD^c^, Coronary heart disease. Q1^d^, magnesium intake is less than or equal to 192.4 mg/day. Q2^e^, magnesium intake ranges from greater than or equal to 192.5 and less than or equal to 252.0 mg/day. Q3^f^, magnesium intake ranges from greater than or equal to 252.1 and less than or equal to 314.4 mg/day. Q4^g^, magnesium intake ranges from greater than or equal to 314.5 and less than or equal to 406.9 mg/day. Q5^h^, magnesium intake ranges from greater than or equal to 407.0 mg/day. *p*-value < 0.05 indicates that the difference is statistically significant.

* Significant difference among magnesium quartiles groups as analyzed by single factor analysis or Kruskal–Wallis test (*p* < 0.05).

### 3.3 Relationship between dietary magnesium intake and depression

A univariate analysis revealed significant associations between depression and age, gender, education level, marital status, family income, BMI, smoking status, hypertension, diabetes, stroke, coronary heart disease, caloric intake, and carbohydrate intake (Table 2).

**Table 2:** Association of covariates and depression risk.

Variable	OR (95% CI)	*p*-value
Age (years)	1 (0.99 ~ 1)	0.005
Sex, *n* (%)		
Male	1 (Ref)	
Female	1.8 (1.66 ~ 1.94)	<0.001^*^
Education level (years), *n* (%)		
≤12	1 (Ref)	
>12	0.57 (0.53 ~ 0.62)	<0.001^*^
Race/Ethnicity, *n* (%)		
Non-Hispanic White	1 (Ref)	
Non-Hispanic Black	1.09 (0.99 ~ 1.2)	0.093
Others	1.03 (0.94 ~ 1.12)	0.564
Marital status, *n* (%)		
Married or living with a partner	1 (Ref)	
Living alone	1.89 (1.75 ~ 2.03)	<0.001^*^
Family income, *n* (%)		
Low	1 (Ref)	
Medium	0.48 (0.45 ~ 0.53)	<0.001^*^
High	0.23 (0.21 ~ 0.26)	<0.001^*^
BMI^a^ (kg/m^2^)	1.03 (1.03 ~ 1.04)	<0.001^*^
Smoking status, *n* (%)		
Never	1 (Ref)	
Current	1.24 (1.13 ~ 1.37)	<0.001^*^
Former	2.64 (2.42 ~ 2.88)	<0.001^*^
Hypertension, *n* (%)		
No	1 (Ref)	
Yes	1.74 (1.62 ~ 1.88)	<0.001^*^
Diabetes, *n* (%)		
No	1 (Ref)	
Yes	1.74 (1.58 ~ 1.9)	<0.001^*^
Stroke, *n* (%)		
No	1 (Ref)	
Yes	2.59 (2.24 ~ 2.99)	<0.001^*^
CHD^b^, *n* (%)		
No	1 (Ref)	
Yes	1.75 (1.5 ~ 2.05)	<0.001^*^
Calorie consumption (kcal/d)	1 (1 ~ 1)	<0.001^*^
Carbohydrate consumption (g/d)	1 (1 ~ 1)	0.009

OR, odds ratio; CI, confidence interval; Ref: reference. BMI^a^, body mass index; CHD^b^, Coronary heart disease. *p*-value < 0.05 indicates that the difference is statistically significant.

* Indicates statistically significant association between the variable and depression risk.

When dietary magnesium consumption was separated into quartiles and potential confounders were controlled for, there was a substantial negative correlation between magnesium intake and depression. In the unadjusted model, the ORs for depression in Q2 (192.5–252.0 mg/day), Q3 (252.1–314.4 mg/day), Q4 (314.5–406.9 mg/day), and Q5 (≥407.0 mg/day) were reduced by 31% (OR = 0.69 [95% CI 0.62, 0.77]), 39% (OR = 0.61 [95% CI 0.54, 0.68]), 54% (OR = 0.46 [95% CI 0.41, 0.52]), and 51% (OR = 0.49 [95% CI 0.43, 0.55]). After adjusting for the variables described in Table 1, the adjusted ORs were 0.83 (95% CI 0.74, 0.93), 0.82 (95% CI 0.72, 0.93), 0.70 (95% CI 0.61, 0.81), and 0.80 (95% CI 0.68, 0.94; *p* < 0.001; Table 3). Using Q4 as the reference group, in the unadjusted model, persons with low dietary magnesium intake had a 64% higher risk of depression (OR = 1.64 [95% CI 1.48, 1.83]), whereas those with high dietary magnesium intake had a 5% higher risk. After adjusting for the variables described in Table 1, the adjusted ORs were 1.23 (95% CI 1.09, 1.38) and 1.16 (95% CI 1.01, 1.34; Table 4).

**Table 3:** Association between dietary magnesium intake and depression.

Variable	Crude	*p*-value	Model 1	*p*-value	Model 2	*p*-value	Model 3	*p*-value
	OR (95% CI)		OR (95% CI)		OR (95% CI)		OR (95% CI)	
Q1^a^ (*n* = 7,019)	1 (Ref)		1 (Ref)		1 (Ref)		1 (Ref)	
Q2^b^ (*n* = 7,011)	0.69 (0.62 ~ 0.77)	<0.001	0.82 (0.74 ~ 0.92)	0.001	0.85 (0.76 ~ 0.95)	0.005	0.83 (0.74 ~ 0.93)	**0.002**
Q3^c^ (*n* = 7,083)	0.61 (0.54 ~ 0.68)	<0.001	0.8 (0.72 ~ 0.9)	<0.001	0.85 (0.76 ~ 0.96)	0.007	0.82 (0.72 ~ 0.93)	**0.002**
Q4^d^ (*n* = 7,074)	0.46 (0.41 ~ 0.52)	<0.001	0.69 (0.61 ~ 0.78)	<0.001	0.74 (0.65 ~ 0.84)	<0.001	0.70 (0.61 ~ 0.81)	**<0.001**
Q5^e^ (*n* = 7,065)	0.49 (0.43 ~ 0.55)	<0.001	0.79 (0.7 ~ 0.9)	<0.001	0.85 (0.75 ~ 0.97)	0.015	0.80 (0.68 ~ 0.94)	**0.006**
Trend test		<0.001		<0.001		0.001		<0.001

Values were calculated using multivariate logistic regression analysis. OR, odds ratio; CI, confidence interval; Ref: reference. Crude: no adjusted. Model 1: adjusted for age + sex + marital status + race/ethnicity + education level + family income. Model 2: Model 1 + smoking status + hypertension + diabetes + stroke. Model 3: Model 2 + CHD + BMI + calorie consumption + carbohydrate consumption. Q1^a^, magnesium intake is less than or equal to 192.4 mg/day. Q2^b^, magnesium intake ranges from greater than or equal to 192.5 and less than or equal to 252.0 mg/day. Q3^c^, magnesium intake ranges from greater than or equal to 252.1 and less than or equal to 314.4 mg/day. Q4^d^, magnesium intake ranges from greater than or equal to 314.5 and less than or equal to 406.9 mg/day. Q5^e^, magnesium intake ranges from greater than or equal to 407.0 mg/day. Bold values: *p*-value < 0.05 indicates that the difference is statistically significant.

**Table 4:** Association between dietary magnesium intake and depression.

Variable	Crude	*p*-value	Model 1	*p*-value	Model 2	*p*-value	Model 3	*p*-value
	OR (95% CI)		OR (95% CI)		OR (95% CI)		OR (95% CI)	
Q1 ~ Q3^a^ (*n* = 21,113)	1.64 (1.48 ~ 1.83)	<0.001	1.27 (1.14 ~ 1.42)	<0.001	1.22 (1.09 ~ 1.36)	<0.001	1.23 (1.09 ~ 1.38)	**<0.001**
Q4^b^ (*n* = 7,074)	1 (Ref)		1 (Ref)		1 (Ref)		1 (Ref)	
Q5^c^ (*n* = 7,065)	1.05 (0.92 ~ 1.21)	0.457	1.15 (1 ~ 1.32)	0.048	1.16 (1.01 ~ 1.33)	0.04	1.16 (1.01 ~ 1.34)	**0.039**
Trend test		<0.001		0.005		0.065		0.118

Values were calculated using multivariate logistic regression analysis. OR, odds ratio; CI, confidence interval; Ref: reference. Crude: no adjusted. Model 1: adjusted for age + sex + marital status + race/ethnicity + education level + family income. Model 2: Model 1 + smoking status + hypertension + diabetes + stroke. Model 3: Model 2 + CHD + BMI + calorie consumption + carbohydrate consumption. Q1 ~ Q3^a^, magnesium intake is less than or equal to 314.4 mg/day. Q4^b^, magnesium intake ranges from greater than or equal to 314.5 and less than or equal to 406.9 mg/day. Q5^c^, magnesium intake ranges from greater than or equal to 407.0 mg/day. Bold values: *p*-value < 0.05 indicates that the difference is statistically significant.

**Figure 2:**
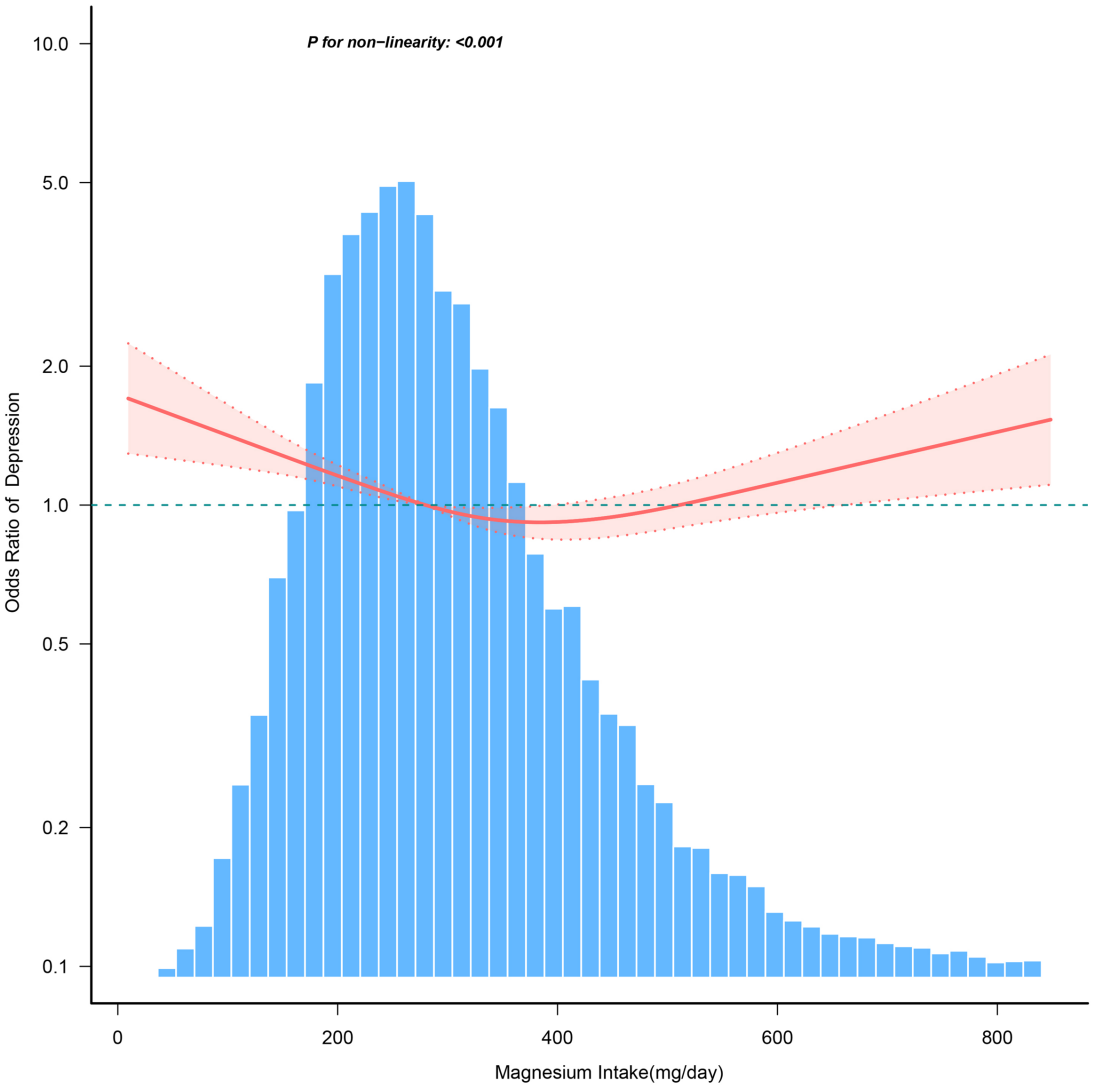
The dose–response relationship between dietary magnesium intake and the risk of depression. Solid and dashed lines represent the predicted value and 95% confidence intervals. They were adjusted for age, sex, race/ethnicity, education level, family income, marital status, smoking status, hypertension, diabetes, stroke, coronary heart disease, body mass index, calorie consumption, and carbohydrate consumption. Only 99% of the data is shown.

After adjusting for various covariates, Figure 2 demonstrates a nonlinear relationship between dietary magnesium intake and the risk of depression. For magnesium intake levels below 366.7 mg/day, each 1 mg increase in magnesium intake is associated with a 0.2% decrease in the risk of depression (OR = 0.998, 95% CI: 0.997–0.999). In contrast, for magnesium intake levels ≥366.7 mg/day, the risk of depression increases by 0.1% for each 1 mg increase in daily magnesium consumption (OR = 1.001, 95% CI: 1.000–1.002; Table 5).

**Table 5:** Threshold effect analysis of the relationship of magnesium intake with depression.

Magnesium Intake, mg/d	Adjusted Model	
	OR (95% CI)	*p*-value
< 366.7	0.998 (0.997 ~ 0.999)	<0.001
≥ 366.7	1.001 (1 ~ 1.002)	0.0069
Log-likelihood ratio test		<0.001

OR, odds ratio; CI, confidence interval. Adjusted Model: adjusted for + age + sex + race/ethnicity + education level + family income + marital status + smoking status + hypertension + diabetes + stroke + CHD + BMI + calorie consumption + carbohydrate consumption. Only 99% of the data is displayed. *p*-value < 0.05 indicates that the difference is statistically significant.

### 3.4 Stratified analyses based on additional variables

**Figure 3:**
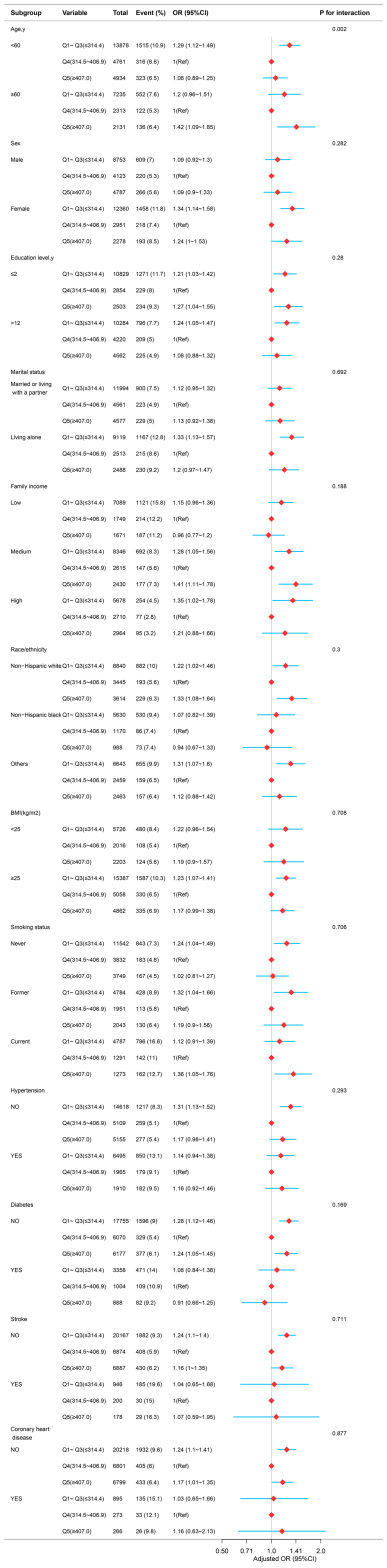
The relationship between dietary magnesium intake and depression in different subgroups. Except for the stratification component itself, each stratification factor was adjusted for all other variables (age, sex, race/ethnicity, education level, family income, marital status, smoking status, hypertension, diabetes, stroke, coronary heart disease, body mass index, calorie consumption, and carbohydrate consumption).

A stratified analysis was performed across multiple subgroups to assess the potential moderating effect of dietary magnesium on the association with depression. After stratifying by age, gender, race, education level, family income, marital status, smoking status, hypertension, diabetes, stroke, coronary heart disease, and body mass index, a significant interaction was observed between dietary magnesium intake and age (*p*-value for the likelihood ratio test for the interaction was *p* = 0.002; Figure 3).

**Figure 4:**
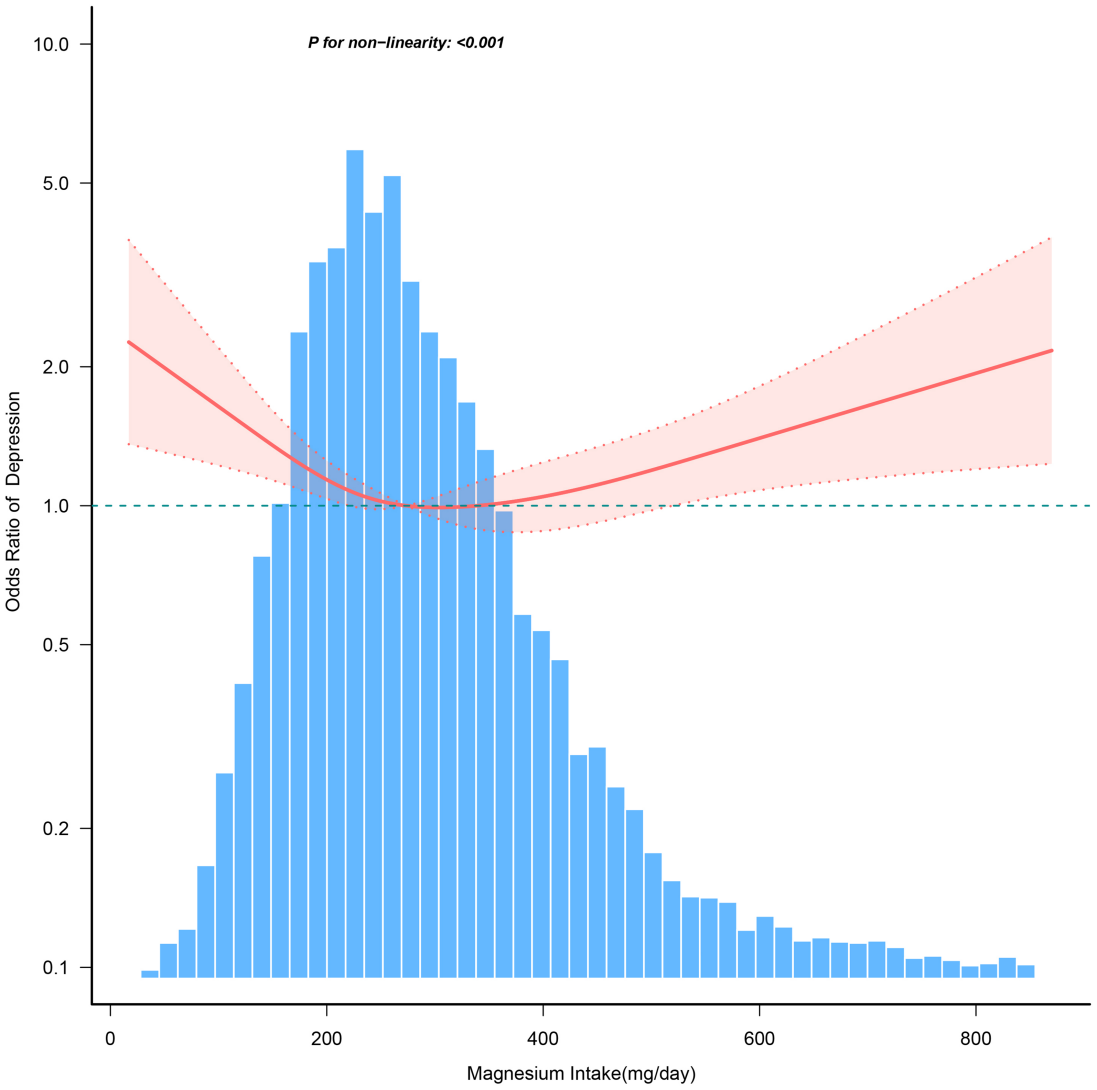
The dose–response relationship between dietary magnesium intake and the risk of depression among individuals over 60. Solid and dashed lines represent the predicted value and 95% confidence intervals. They were adjusted for age, sex, race/ethnicity, education level, family income, marital status, smoking status, hypertension, diabetes, stroke, coronary heart disease, body mass index, calorie consumption, and carbohydrate consumption. Only 99% of the data is shown.

Figure 4 illustrates a nonlinear relationship between magnesium consumption and depression in individuals aged ≥60 years (*p* < 0.001). And the link is inverse L-shaped. For individuals with magnesium intake ≥270.7 mg/day, each 1 mg/day increase in magnesium consumption is associated with a 0.1% increase in the incidence of depression (Table 6).

**Table 6:** Threshold effect analysis of the relationship of magnesium intake with depression among individuals over 60.

Magnesium Intake, mg/d	Adjusted Model	
	OR (95% CI)	*p*-value
<270.7	0.998 (0.996 ~ 1)	0.0817
≥270.7	1.001 (1 ~ 1.002)	0.0132
Log-likelihood ratio test		<0.001

OR, odds ratio; CI, confidence interval. Adjusted Model: adjusted for sex + race/ethnicity + education level + family income + marital status + smoking status + hypertension + diabetes + stroke + CHD + BMI + calorie consumption + carbohydrate consumption. Only 99% of the data is displayed. *p*-value < 0.05 indicates that the difference is statistically significant.

### 3.5 Sensitivity analysis

After excluding 412 individuals with excessive calorie intake, the relationship between dietary magnesium intake and depression remained unchanged. When compared to the Q4 group (314.5–406.9 mg/day), the adjusted odds ratios for depression were 1.23 (95% CI: 1.09–1.38, *p* = 0.001) for participants in Q1–Q3 (≤314.4 mg/day) and 1.16 (95% CI: 1.00–1.33, *p* = 0.049) for participants in Q5 (≥407.0 mg/day; Table 7).

**Table 7:** Association between dietary magnesium intake and depression among individuals with a daily caloric intake between 500 and 5,000 kcal.

Variable	Crude	*p-*value	Model 1	*p*-value	Model 2	*p*-value	Model 3	*p*-value
	OR (95% CI)		OR (95% CI)		OR (95% CI)		OR (95% CI)	
Q1 ~ Q3^a^ (*n* = 20,964)	1.64 (1.47 ~ 1.82)	<0.001	1.27 (1.13 ~ 1.41)	<0.001	1.22 (1.09 ~ 1.36)	0.001	1.23 (1.09 ~ 1.38)	**0.001**
Q4^b^ (*n* = 7,063)	1 (Ref)		1 (Ref)		1 (Ref)		1 (Ref)	
Q5^c^(*n* = 6,813)	1.03 (0.9 ~ 1.19)	0.632	1.14 (0.99 ~ 1.31)	0.063	1.15 (1 ~ 1.33)	0.048	1.16 (1 ~ 1.33)	**0.049**
Trend test		<0.001		0.004		0.059		0.104

Values were calculated using multivariate logistic regression analysis. OR, odds ratio; CI, confidence interval; Ref: reference. Crude: no adjusted. Model 1: adjusted for age + sex + marital status + race/ethnicity + education level + family income. Model 2: Model 1 + smoking status + hypertension + diabetes + stroke. Model 3: Model 2 + CHD + BMI + calorie consumption + carbohydrate consumption. Q1 ~ Q3^a^, magnesium intake is less than or equal to 314.4 mg/day. Q4^b^, magnesium intake ranges from greater than or equal to 314.5 and less than or equal to 406.9 mg/day. Q5^c^, magnesium intake ranges from greater than or equal to 407.0 mg/day. Bold values: *p*-value < 0.05 indicates that the difference is statistically significant.

## 4 Discussion

This large retrospective cross-sectional research study of US adults found a substantial, nonlinear relationship between magnesium consumption and depression risk, along with a nonlinear dose–response curve. Moderate magnesium intake was protective, whereas low and high intake levels increased the risk of depression. In addition, age emerged as a key moderator in this relationship. Higher magnesium consumption has been associated to an increased risk of depression in adults aged 60 and up. These findings highlight the complex function of magnesium in mental health, implying that focused dietary interventions may need to account for age-related physiological variations. This is especially important for East Asian communities, as magnesium intake is often lower than in Caucasian populations, potentially impacting depression prevention efforts in these countries.

Our findings align partially with prior studies suggesting the protective role of magnesium against depression but differ in detailing the nonlinear dose–response relationship. For instance, Chou et al. (24) reported no association between dietary magnesium and depressive symptoms in a Taiwanese cohort. Similarly, a prospective study among Spanish university graduates found no significant relationship (23). These discrepancies could stem from differences in study design, dietary assessment methods, or population characteristics. A review (27) found that magnesium shortage may increase vulnerability to stress. Some studies (28, 29) have showed that insufficient dietary magnesium is a primary cause of treatment-resistant depression (TRD), and magnesium supplementation may prevent depression and serve as an additional treatment. More research is needed to validate our findings, explore the detailed correlations, and identify potential processes.

The precise processes by which magnesium effects mental diseases remain unknown. However, numerous routes are thought to contribute to its effects on depression. Magnesium is essential for brain function, stress response control, and neurotransmission (30, 31). Magnesium ions play a crucial role in regulating glutamate transmission by affecting the activation of NMDA receptors (12). Magnesium shortage may result in overactivation of NMDA receptors, causing neurotoxicity and raising the risk of depression. Magnesium plays a crucial role in neurotransmitter control, affecting serotonin and dopamine synthesis and release, improving neuronal function, and preventing excitotoxicity, all of which impact mood and cognition (13). Magnesium offers anti-inflammatory properties. It reduces the secretion of pro-inflammatory cytokines such IL-6 and TNF-α (31), which can alleviate chronic inflammation. Chronic inflammation is regarded as a possible pathogenic cause for depression. Recent animal investigations suggest that magnesium deprivation can disrupt gut-brain axis communication and impact mood regulation (14).

Our study indicated that persons aged ≥60 years have a higher risk of depression with increased dietary magnesium intake. These data are congruent with those of Tarleton et al. (21) and it emphasizes the crucial moderating effect of age on the association between dietary magnesium consumption and depression risk. This interaction mechanism is believed to involve several components. To begin, as individuals age, their metabolic function and mineral absorption capabilities alter (32–34), potentially resulting in variable magnesium requirements and utilization efficiency in older adults compared to younger populations (35–38). Furthermore, older persons may have more chronic conditions and use more drugs (39–41), which may affect magnesium metabolism and its effect on the neurological system. Excess magnesium intake in older individuals may result in negative health effects, such as arrhythmias or gastrointestinal discomfort (42–44), indirectly influencing mental health. This finding is consistent with our findings from the nonlinear association study, which showed that moderate magnesium consumption protects against depression risk, whereas high magnesium intake may raise the risk. Therefore, nutritional recommendations should be adjusted to different age groups.

Our study also has limitations. First, while this study is cross-sectional, we cannot demonstrate a causal relationship between dietary magnesium intake and depression. Additional well-designed cohort studies are required to resolve this shortcoming. Second, self-reported dietary intake data may include recollection bias, reducing the precision of the findings. Finally, residual confounding factors, such as genetic predispositions or other lifestyle behaviors, may contribute to the risk of depression but were not adequately accounted for in this study.

## 5 Conclusion

This study demonstrated a complex relationship between dietary magnesium intake and the risk of depression, with age playing an important role. These findings provide significant scientific information for future nutritional therapies and depression prevention efforts. The results also provide possibilities for future research into the mechanisms of magnesium impact in various groups.

## Data Availability

The raw data supporting the conclusions of this article will be made available by the authors, without undue reservation.

## Footnotes

^1^ http://www.cdc.gov/nchs/nhanes.htm

^2^ http://www.R~project.org
